# Unravelling the complexity of the interactions among VACCIMEL, BCG and blood monocytes

**DOI:** 10.3389/fimmu.2026.1731270

**Published:** 2026-02-26

**Authors:** Erika Schwab, Brenda Carles, Alicia Inés Bravo, María Victoria Echenique, Agustina De Franc, Mariano Guillermo Bonanno, María Marcela Barrio, José Mordoh

**Affiliations:** 1 Facultad de Ciencias Exactas y Naturales, Universidad de Buenos Aires, Buenos Aires, Argentina; 2 Centro de Investigaciones Oncológicas-Fundación Cáncer (FUCA), Buenos Aires, Argentina; 3 Hospital Interzonal General de Agudos (HIGA) Eva Perón, San Martín, Argentina; 4 LANAIS-MIE, Instituto de Biología Celular y Neurociencia Prof. E. De Robertis, Facultad de Medicina, Universidad de Buenos Aires, Buenos Aires, Argentina

**Keywords:** BCG, cutaneous melanoma, immunotherapy, innate immune cells, monocytes, phagocytosis

## Abstract

VACCIMEL is an immunotherapeutic adjuvant treatment for high-risk cutaneous melanoma. It consists of 13 intradermal injections of four irradiated melanoma cell lines co-adjuvated with BCG and GM-CSF. VACCIMEL prolongs distant metastases-free survival and it induces T lymphocytes reactive against melanoma differentiation antigens, cancer testis antigens, and neoantigens. In this paper, we have studied *in vitro* the interaction among VACCIMEL, BCG, and blood-derived monocytes, a fundamental component of innate immunity. We have demonstrated that monocytes may phagocytose, separately and jointly, BCG and VACCIMEL. We have shown for the first time by transmission electron microscopy, detailed features of irradiated melanoma cells phagocytosis and its timeline. We have equally demonstrated that monocytes may process and cross-present tumor antigens to CD8^+^ T cells and that BCG interferes with that process in a multiplicity of infection (MOI) - dependent manner. BCG induces high production of IL-10 by monocytes which is several-fold reduced by phagocytosis of tumor antigens. Although cross-presentation is still inhibited in the presence of a high BCG MOI (0.4), it rebounds by reducing tenfold the BCG MOI from 0.4 to 0.04. This suggests that an adequate balance between tumor antigens and BCG phagocytosis is needed to retain the stimulatory properties of activated monocytes and trigger immunogenicity of tumor antigens.

## Introduction

Since the successful introduction of immunotherapy with anti-checkpoint antibodies as a treatment for cutaneous melanoma the prognosis of patients has considerably improved (1). However, meaningful clinical responses only occur in a subset of patients, and further improvements of immunotherapy are necessary. As a consequence, a combination of different strategies are being explored. VACCIMEL is an adjuvant immunotherapeutic treatment, composed of four irradiated allogeneic human cutaneous melanoma cell lines to which Bacillus Calmette-Guerin (BCG) and granulocyte-macrophage colony-stimulating factor (GM-CSF) are added. VACCIMEL provides multiple cutaneous melanoma antigens; BCG would help to create an inflammatory milieu through the release of proinflammatory cytokines (2) and GM-CSF would provide increased vascularization and would facilitate interaction between lymphocytes and myeloid cells at the vaccination site (3, 4). VACCIMEL was studied in a Phase I clinical study on stages IIB–IV cutaneous melanoma patients to determine its toxicity and the optimal dose of GM-CSF (5). Another Phase I clinical trial was performed on Stage IIB-IV cutaneous melanoma patients, to whom blood monocyte-derived, mature dendritic cells (DC), loaded with VACCIMEL, were administered (6). In both Phase I studies, 70% of Stage IIB and III patients attained long survival times without developing metastatic disease, and serious adverse events (grades 3 and 4) were not observed. Subsequently, a phase II randomized clinical study was undertaken, in which stages IIB, IIC, and III cutaneous melanoma patients were randomized to receive either VACCIMEL or medium-dose IFN-alpha 2b. In that study, VACCIMEL was more efficient than IFN-alpha 2b in prolonging distant metastasis-free survival (7).

Several studies from our group (8–10) demonstrated that VACCIMEL elicited a polyclonal cellular immune response against melanocytic differentiation antigens (MDA), cancer-testis antigens, and neoantigens derived both from the patient´s tumor and from VACCIMEL. Therefore, a broad immunization process was taking place. However, a detailed knowledge of the immune events that occurred at the inoculation site at the beginning of treatment with VACCIMEL and its adjuvants is lacking. The study of the vaccination sites in humans is not abundant. Melssen et al. have made a longitudinal analysis of the vaccination sites in two groups of patients with cutaneous melanoma in whom Incomplete Freund´s adjuvant (IFA) or AS15 (a combination of TLR4 and TLR9 agonists with QS21) were used as adjuvants, but they mainly focused on the lymphocyte and DC composition of the infiltrate; monocytes were not studied in that work (11). Also, Schaffer et al. studied the histological composition of inoculation sites after vaccination with IFA alone or melanoma peptides; also, here, monocytes were not studied, and no histological differences were found attributable to peptides (12). Since systematic access to vaccination sites by biopsies in humans are difficult to obtain for ethical reasons, the interaction between monocytes obtained from healthy donors (HD) with VACCIMEL components was studied *in vitro* as a model of their encounter at the inoculation site.

Previous to analyzing the immunization process, the expression of immunogenic molecules after thawing the irradiated VACCIMEL cells, events that in patients takes place after vaccination, had to be determined. After that, the interaction of VACCIMEL with blood monocytes (which will be thereafter referred to as monocytes) was first analyzed since, besides neutrophils and resident macrophages, they are among the immune cells that promptly arrive at sites in which an infection or inflammatory process has been triggered, and have demonstrated considerable plasticity in their responses (13, 14). In VACCIMEL immunotherapy, two elements that may be phagocytosed are present: VACCIMEL and BCG. We investigated the kinetics of VACCIMEL and BCG phagocytosis by monocytes immunohistochemistry and electron microscopy, and monocytes differentiation by flow cytometry. Monocyte’s ability to cross-present a VACCIMEL–derived antigen to cognate T cells was also evaluated. It was found that: i) irradiated VACCIMEL disintegrated over the course of several days, while retaining markers expression; ii) monocytes were able to simultaneously phagocytose BCG and VACCIMEL components; iii) BCG altered monocytes differentiation, with a stabilization of the classical phenotype for at least the first 48 h of culture; iv) monocytes could cross-present PMEL/gp100 MDA antigen to CD8^+^ T lymphocytes, although BCG diminished cross-presentation, in a multiplicity of infection (MOI)-dependent manner.

## Results

### Fate of irradiated VACCIMEL cells and tumor markers expression after thawing

**Figure 1:**
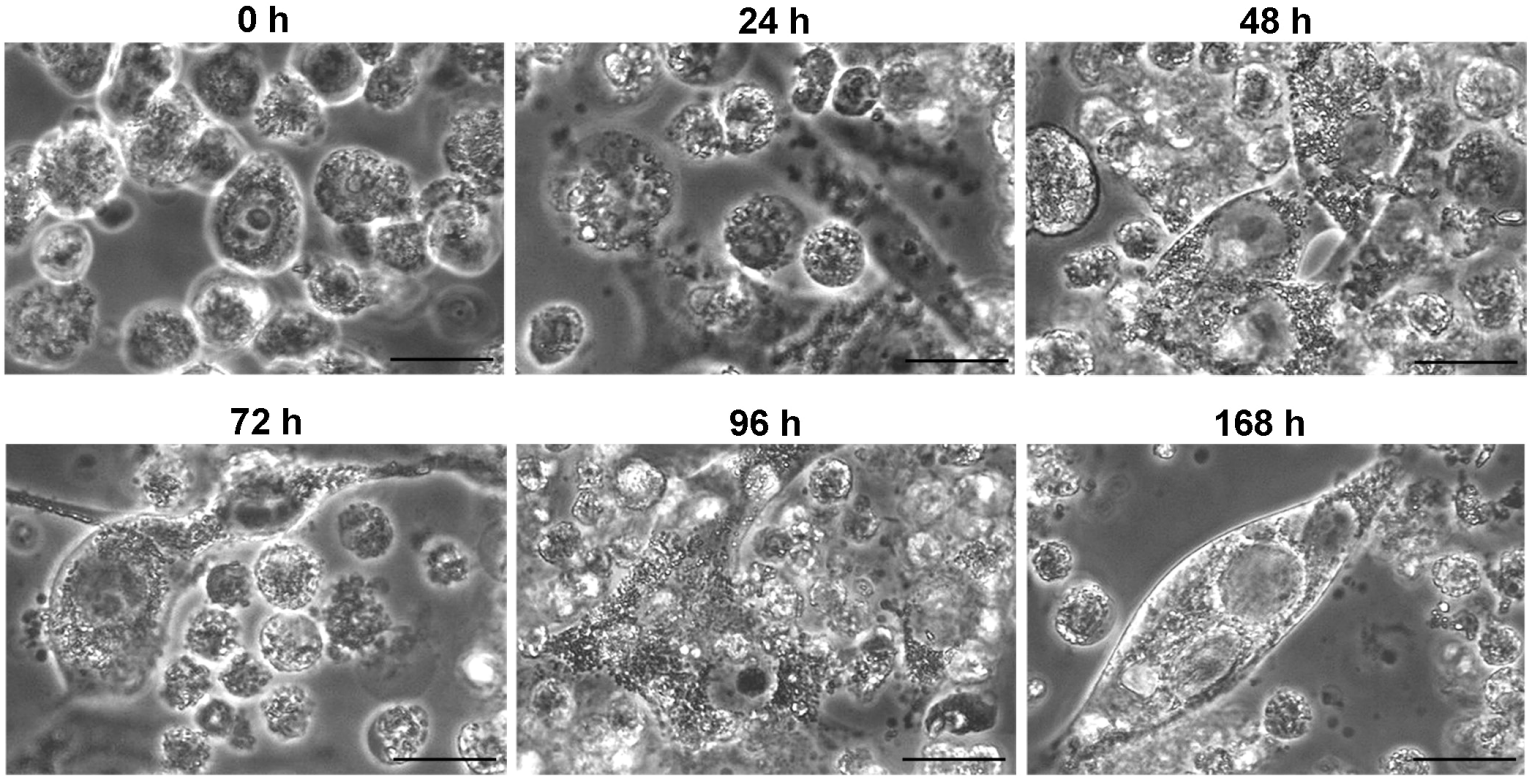
Morphological changes of VACCIMEL over time after irradiation, freezing, and thawing. Representative pictures are shown at the corresponding times after VACCIMEL thawing. Observation under phase contrast microscopy (original magnification: 400X; scale bar= 10 µm).

VACCIMEL is a mixture of four 70 Gy-irradiated allogeneic cutaneous melanoma cell lines, which are injected intradermally in patients, not later than 3 h after thawing. To investigate the fate of the thawed cells over time, we observed their morphology up to 168 h in tissue culture. The results are shown in **Figure 1**. Immediately after thawing, the cells appeared intact, with dense cytoplasm. Most cells were refringent, suggesting an intact cell membrane, whereas some had lost refringence, indicating altered permeability. Vacuoles were observed beneath the cell membrane. This pattern was altered after 24 h since most remaining cells were non-refringent, vacuoles appeared in the cytoplasm, and some cell membranes were ruffled, indicating a process of apoptosis; only 13.7% of the cells remained intact. Many cells appeared loaded with vesicles, some of which were described as Radiation Induced Lipid Bodies, and their lipid composition has been described (15). After 48 h, the vacuoles increased in the cytoplasm of the remaining cells, and some cell fusions started to be noticed; the number of non-refringent cells increased. This process intensified after 72 h and 96 h, and fused cells with bizarre forms were more abundant. After 168 h, only a few fused cellular masses were observed. Therefore, even as soon as 24 h after thawing, a process of cellular apoptosis and necrosis appeared, which increased with time. The total cell number and integrity decreased significantly during the first 48 h, with only 39.4% of the starting cell number persisting, of which 8.1% retained integrity as analyzed by generalized linear mixed models (GLMMs) with gamma errors (**Table 1A**).

**Table 1:** Evolution of thawed VACCIMEL over time.

A						
Time (h)	Total cell Number mean x10^6^ (% ± SD)			Cell integrity mean x10^6^ (% ± SD)		
0	16.9 (100)			6.2 (36.6 ± 1.3)		
24	8.5 (50 ± 1.9)			1.2 (13.7 ± 1.6)		
48	6.7 (39.4 ± 4.6)			0.6 (8.1 ± 1.1)		

B						
Sample	Time (h)	Ki67 (%)	HLA-I (%)	PMEL/gp100 (%)	MART-1 (%)	MAGEA1 (%)
Non-irradiated VACCIMEL cells	-	62.7 ± 8	59.5 ± 7.6	45.7 ± 9.9	55.7 ± 8.6	28.8 ± 14.2
VACCIMEL (time after thawing)	0	69.6 ± 6.4	46.1 ± 14	36.3 ± 10.4	37.7 ± 13.4	35.9 ± 6.8
	24	43.9 ± 5.9	45 ± 5.9	42.9 ± 5.6	47.7 ± 6.2	25.9 ± 11.9
	48	69.7 ± 27.2	45.6 ± 6.2	43.9 ± 6.6	39.9 ± 4.5	28.2 ± 4.5

A. Total cell number and integrity, as measured with trypan blue, were determined at different times after thawing (n=2). Differences in total cell number and integrity were statistically significant between 0–24 h,0–48 h and 24–48 h (all p<0.0001) as analyzed using GLMMs with gamma errors, as described under Materials and Methods. B. At different times after thawing, immunohistochemistry of different markers was determined as described under Materials and Methods. The percentage expression of each marker was determined by counting 10 fields at 1000× magnification; mean percentage ± SD is shown. Non-significant differences in expression of Ki67, HLA-I, PMEL/gp100, MART-1 and MAGEA1 were found between 0 and 48 h post-thawing using GLMs with binomial errors distribution.

**Figure 2:**
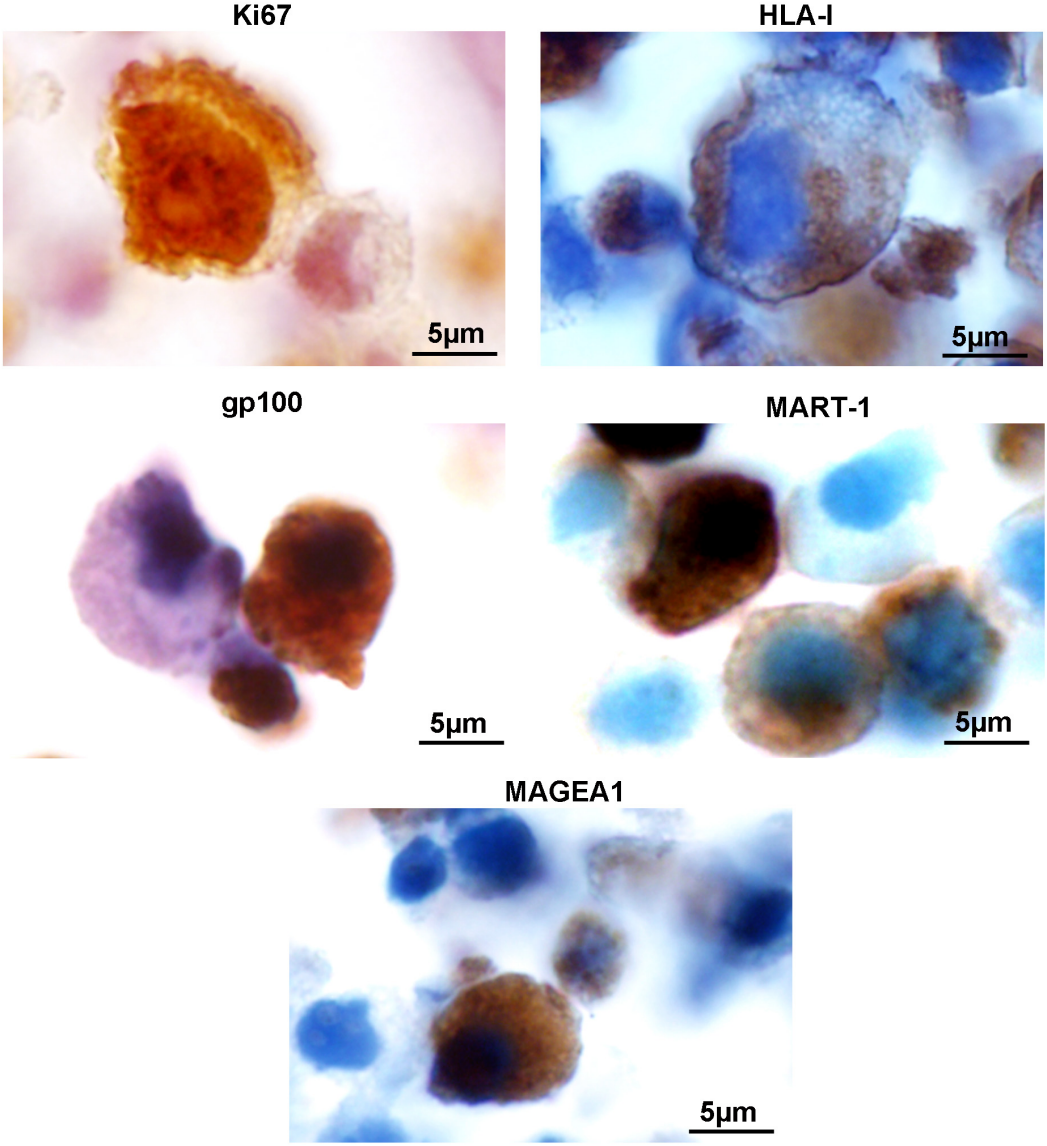
VACCIMEL expression of Ki67, HLA-I, PMEL/gp100, MelanA/MART-1, and MAGEA1 after thawing. VACCIMEL cells were cultured for 48 h, and immunohistochemical staining was performed as described under Materials and Methods. Representative pictures are shown (original magnification: 1000×; scale bar = 5 µm).

The persistence of some relevant markers was determined by immunohistochemistry: i) Ki67 to measure residual DNA synthesis and nuclear integrity; ii) HLA-I to determine the fate of this allogeneic marker; iii) PMEL/gp100 and MelanA/MART-1 as MDA; and iv) MAGEA1 antigen, representative of cancer testis antigens (**Table 1B**, **Figure 2**). The expression of these markers did not change significantly up to 48 h from thawing as analyzed by generalized linear models (GLMs), with binomial errors. Examples of the immunohistochemical appearance of these markers are shown in **Figure 2**. It was noteworthy that 48 h after thawing, Ki67 staining was not restricted to the nuclei, since it was also observed in the cytoplasm and in the vicinity of the cell membrane, suggesting a loss of nuclear membrane integrity (**Figure 2**). Therefore, a passage of DNA into the cytoplasm and its eventual release into the extracellular milieu is suggested. Thus, it was found that although the number of irradiated cells diminished over time, their antigen expression remained relatively constant, suggesting that the cellular antigenic offer to the immune system persisted for several days after inoculation. Also, these results imply that the antigenic material that was released into the extracellular milieu increased at least during the first 48 h after inoculation.

### Monocytes phagocytose VACCIMEL

During vaccination, each VACCIMEL dose (1.6x10^7^ irradiated allogeneic melanoma cells) is injected i.d. (intradermal) along with 120 µg protein BCG (~2–10 x10^5^ colony forming units) and 400 µg rhGM-CSF (divided in four 100 µg/day i.d. injections at the vaccination site). Once VACCIMEL is injected into the dermis in conjunction with BCG and GM-CSF, monocytes are among the first innate immune cells arriving at the injection site (16). We investigated the *in vitro* uptake of PKH67–labelled VACCIMEL by monocytes obtained from healthy donors (HD), either in the presence or absence of BCG at two different MOI, and 100 ng/ml GM-CSF during co-culture. It is difficult to calculate the actual amount of BCG and GM-CSF to which monocytes are exposed *in vivo* after the first and successive vaccinations, since it would be a balance between the amount of injected BCG and BCG clearance, which has been demonstrated to be also quite efficiently carried out by neutrophils (17) and the number of recruited monocytes at the inoculation site. For the *in vitro* assays we have chosen a 15µg/ml protein BCG dose (MOI 0.04 BCG: monocyte), taking as a reference a similar BCG concentration that has been previously shown to be active in trained immunity *in vitro* assays (18); we also tested a 10 x dose (MOI 0.4 BCG: monocyte).

**Figure 3:**
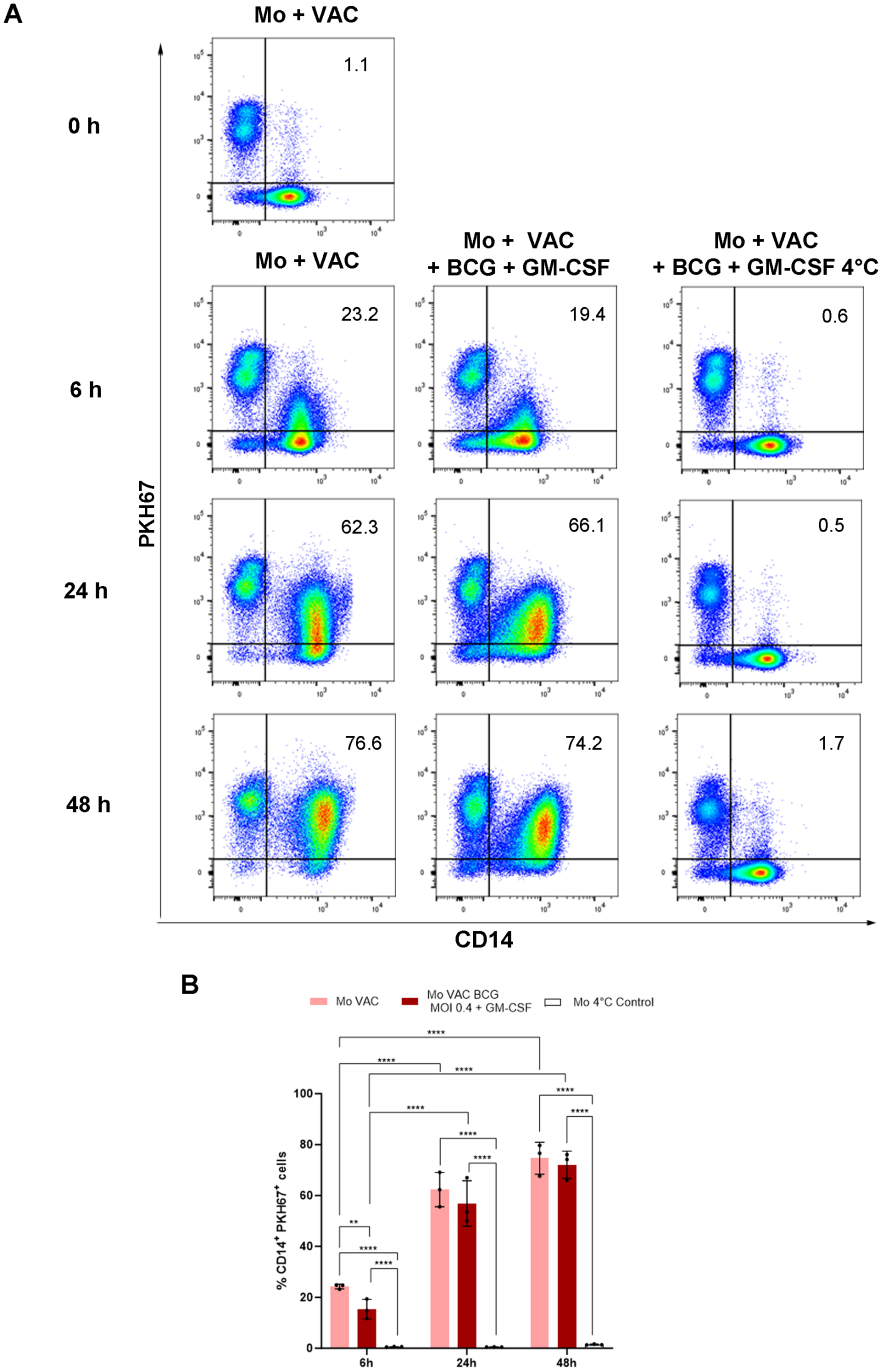
VACCIMEL uptake by monocytes. VACCIMEL cells (VAC) were labelled with PKH67, and co-incubated for 0, 24, and 48 h with purified monocytes (Mo) in the presence or absence of BCG + GM-CSF and analyzed with flow cytometry as described under Materials and Methods. **(A)** Plots from one representative experiment performed at BCG to monocyte MOI 0.4 are shown. Double-labelled CD14^+^PKH67^+^ cells identify monocytes that have incorporated VACCIMEL material. **(B)** Quantitation of VACCIMEL uptake by monocytes is shown as the mean ± SD percentage of CD14^+^PKH67^+^ population after the indicated times (n=3). For each time point, a control culture was performed at 4°C to inhibit phagocytosis. Comparisons were made for each experimental condition between 6–24 h, 6–48 h, and 24–48 h. Also, comparisons were made between the three experimental conditions at each time point. **0<0.01, and ****p<0.0001 (GLMMs, with Gaussian errors).

Viable VACCIMEL cells were labeled with PKH67 and monocytes were purified as described under Materials and Methods. After irradiation, freezing, and thawing, their uptake by monocytes was evaluated by flow cytometry and transmission electron microscopy. For every co-culture experiment, the single-cell populations were analyzed from the monocytes gate to exclude cell clumps (doublets), as observed in the flow cytometry strategy (**Supplementary Figure 1**). This analysis revealed that after coincubation, progressive amounts of PKH67-labelled VACCIMEL material were incorporated by CD14^+^ cells (**Figure 3A**). When the assay was performed using purified monocytes from different HD (n=3) (**Figure 3B**), significant increases of the percentages of double labelled CD14^+^PKH67^+^ cells were found from 6 to 24, and 48 h in the presence and in the absence of BCG (MOI 0.4) plus GM-CSF (p<0.05, GLMMs with Gaussian errors). At 6 h, PKH67-labelled VACCIMEL phagocytosis was significantly higher in the absence of BCG (MOI 0.4) plus GM-CSF (p= 0.0022) than in its presence, but this difference was lost at longer times. After 48 h of co-culture, phagocytosis reached a mean of 74.6% in the absence of BCG and 72.1% in the presence of BCG (MOI 0.4) plus GM-CSF, respectively. At each time point, a control culture performed at 4°C significantly inhibited PKH67-labelled VACCIMEL phagocytosis by monocytes at all the times assayed (p<0.0001). Similar results were obtained when BCG was assayed at MOI 0.04 plus GM-CSF (not shown). Thus, monocytes were able to phagocytose VACCIMEL equally, either in the presence or absence of BCG plus GM-CSF.

**Figure 4:**
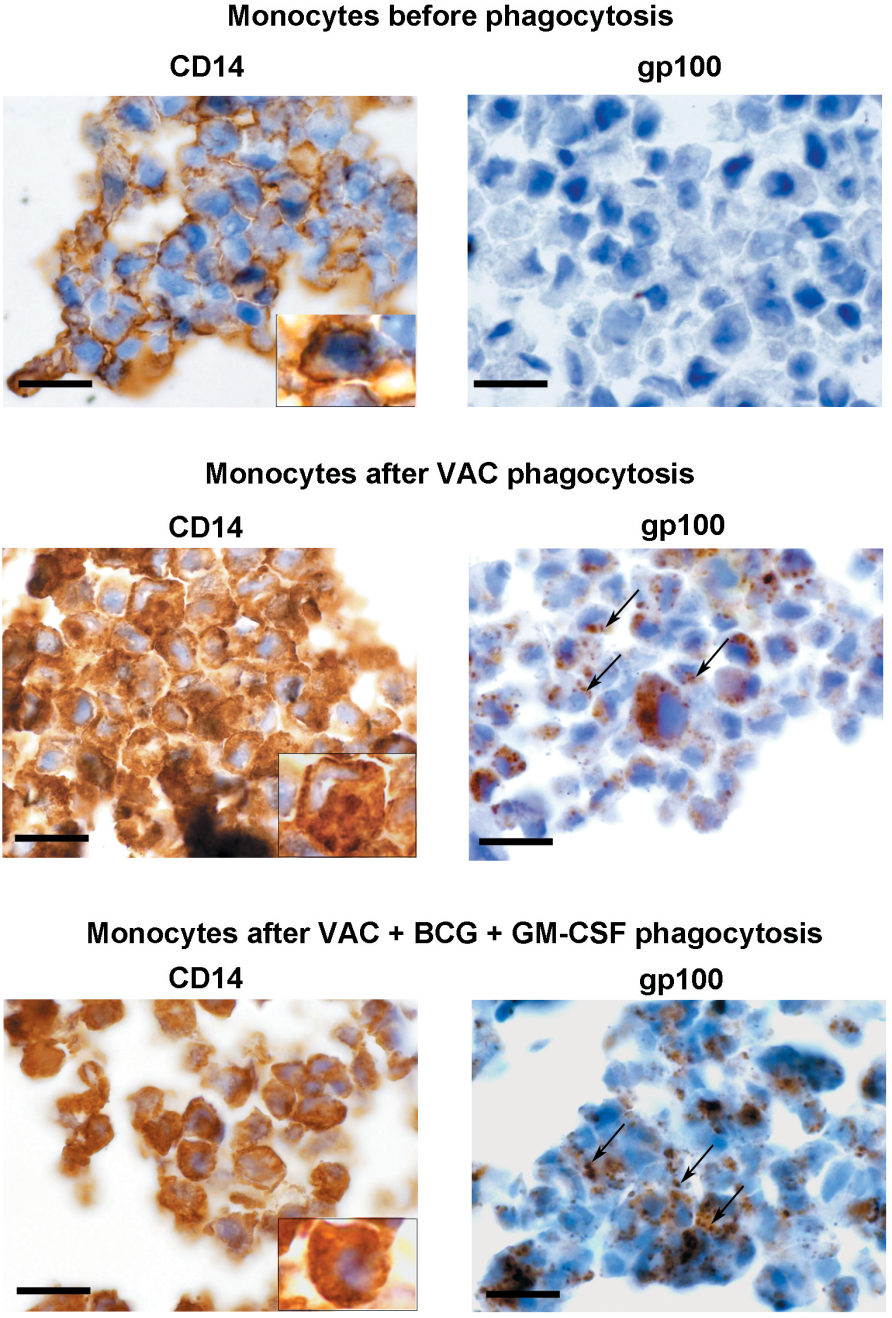
Monocytes capture of PMEL/gp100 antigen from VACCIMEL. Monocytes were analyzed by immunohistochemistry before and after VACCIMEL phagocytosis (48 h) in the presence or absence of BCG plus GM-CSF, using anti-CD14 and anti-PMEL/gp100 antibodies, as described under Materials and Methods. In the insets, monocyte CD14 membrane expression before phagocytosis and intracellular staining after phagocytosis can be observed in detail. Arrows indicate the presence of PMEL/gp100^+^ structures inside monocytes after VACCIMEL phagocytosis either with or without BCG. Original magnification: 1000×; scale bar= 10 µm.

To investigate how long the phagocytosed material took to be digested, we asked if PMEL/gp100, a highly immunogenic MDA expressed in ~40% of VACCIMEL cells, could still be detected by immunohistochemistry in monocytes which would mean that the immunogenic peptide had not yet been digested. After 48 h co-culture, monocytes were purified by CD14^+^ affinity columns to eliminate remaining VACCIMEL cells. Purified monocytes were fixed, paraffin-embedded, and processed for immunohistochemistry for CD14 and PMEL/gp100. As observed in **Figure 4**, the PMEL/gp100 epitope could be detected inside purified monocytes, cultured both in the presence and absence of BCG plus GM-CSF. This result implies that antigenic material from VACCIMEL incorporated into monocytes is incompletely digested after 48 h, a finding that is supported by cross-presentation analysis (see below). It is interesting to observe that CD14, which in baseline monocytes is restricted to the cell membrane, after 48 h phagocytosis may be clearly observed in the cytoplasm, accompanying VACCIMEL phagocytosis (**Figure 4**).

### Monocytes capture and digestion of VACCIMEL and BCG as observed by transmission electron microscopy

**Figure 5:**
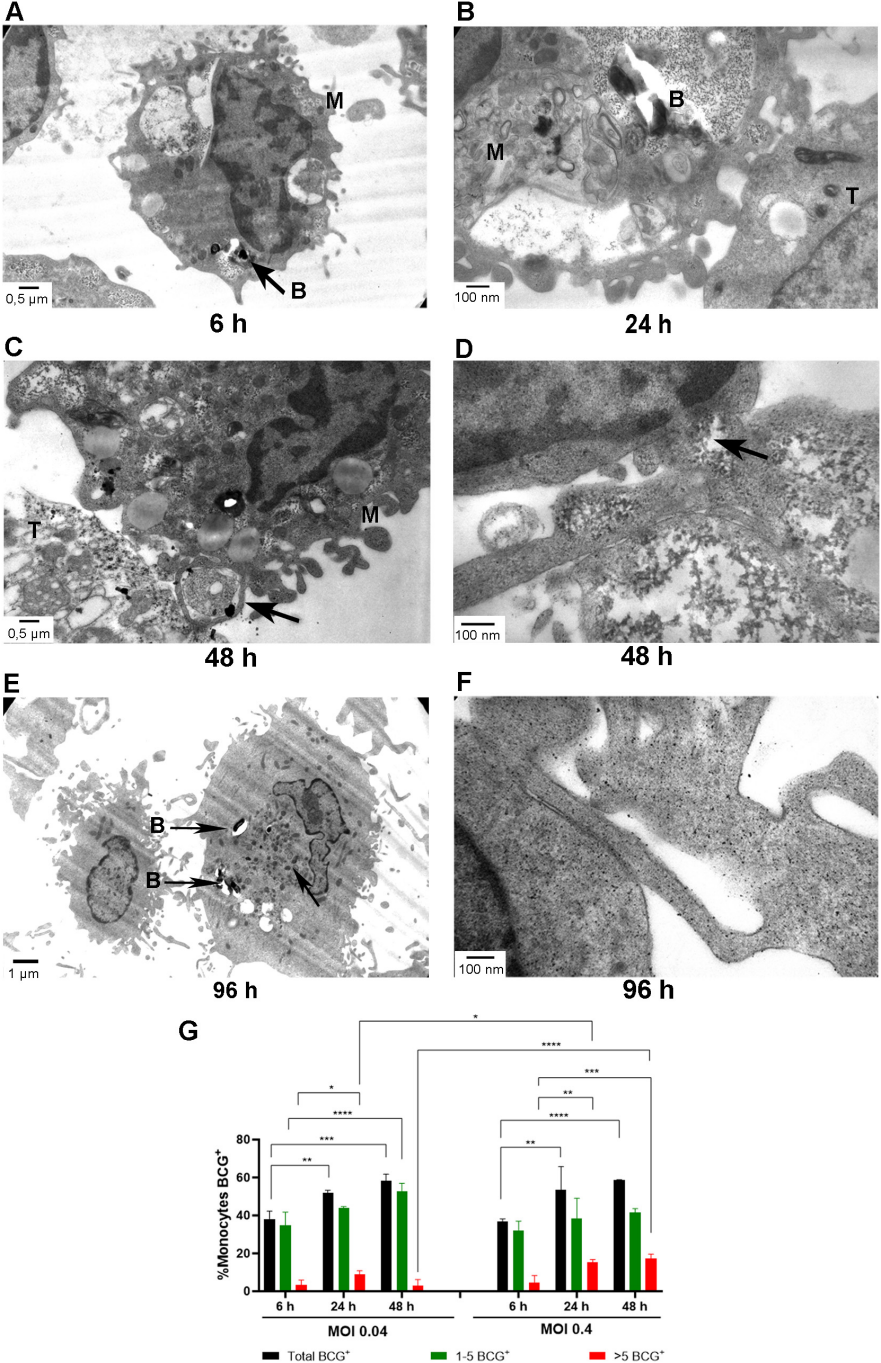
Transmission electron microscopy of monocytes + VACCIMEL plus BCG and GM-CSF co-cultures. Monocytes were co-cultured with VACCIMEL + BCG (MOI 0.4 or 0.04) and GM-CSF for the indicated times, fixed with 4% glutaraldehyde, and processed for transmission electron microscopy as described under Materials and Methods. **(A-F)** Pictures obtained at MOI 0.4 are shown. Magnification: **(A, C)** = 12,000X, scale bar: 0.5 µm; **(B, D, F)** = 50,000X, scale bar: 100 nm; **(E)** = 7,000X, scale bar: 1 µm. T= tumor cell; B= BCG; M= monocyte. **(G)** Monocytes that incorporated BCG after 6, 24, and 48 h incubation were quantified from the transmission electron microscopy pictures as described under Materials and Methods. For MOI = 0.04, 921 monocytes and for MOI 0.4, 653 cells were counted respectively. Results are shown as percentage of monocytes containing BCG (monocytes BCG^+^), either total (Total BCG^+^) or separated into two groups: 1–5 BCG^+^ per monocyte, and >5 BCG^+^ per monocyte (n=2). Results were analyzed by GLMMs with binomial errors distribution. Comparisons were made for each BCG MOI between 6–24 h, 6–48 h and 24–48 h for each group. Also, comparisons were made between BCG MOI 0.4 and 0.04, at each time point. *p<0.05; **p<0.01: ***p<0.001; ****p<0.0001.

To our knowledge, for the first time, the combined phagocytosis of tumor cells and BCG by monocytes was analyzed by transmission electron microscopy. VACCIMEL cells were easily recognized by their smooth cell membranes, pale and sometimes disintegrated cytoplasm. In some cells, melanosomes were detected. Scarce mitochondria were observed. Monocytes may be recognized by their horseshoe-shaped nuclei with heterochromatin attached to the nuclear membrane. In **Figures 5A**, after 6 h of co-incubation, a monocyte presenting phagolysosomes containing one to several BCG is observed. In **Figures 5B**, after 24 h co-culture, a tumor cell and a monocyte may be observed in contact; the coexistence within phagolysosomes of BCG and amorphous material was observed. In **Figure 5C, D** two types of contacts may be observed between monocytes and tumor cells: pseudopodia (**Figure 5C**), which capture particulate material, and membrane bulges in monocytes making contact with tumor cells, allowing passage of particulate material between both cells (**Figure 5D**). In **Figure 5E** a mature monocyte after 96 h in culture is observed, with many lysosomes in the cytoplasm. In **Figures 5F**, a monocyte pseudopod is observed making contact with a tumor cell and through which small particles about 100 nm in diameter may be seen flowing between both cells. BCG uptake by monocytes was quantified from the transmission electron microscopy images and showed that the percentage of monocytes that ingested BCG bacilli increased significantly with the incubation time from 6 to 48 h reaching around 60% (**Figure 5G**). At 48 h, a significantly higher proportion of monocytes that incorporated >5 bacilli per cell was found after phagocytosis of BCG at MOI = 0.4 as compared to MOI = 0.04 (p<0.0001) as analyzed by GLMMs assuming Gaussian errors.

### BCG plus GM-CSF, but not VACCIMEL, alter monocytes phenotype

**Figure 6:**
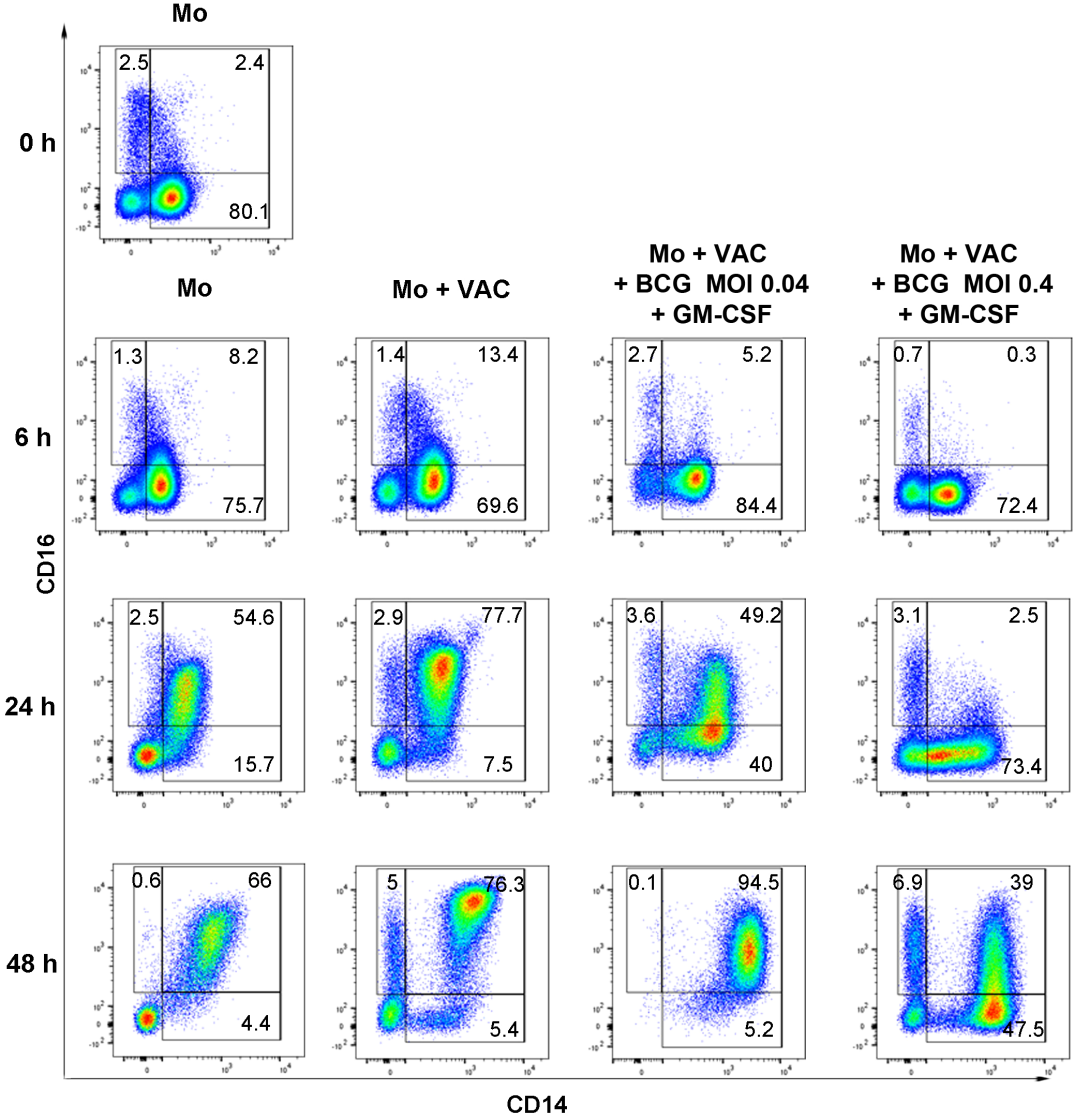
Immunophenotyping of monocytes after co-culture with VACCIMEL, BCG, and GM-CSF. Monocytes (Mo) were incubated for the indicated times, and their phenotype was analyzed by flow cytometry after labeling of CD14 and CD16 markers as described under Materials and Methods. Four independent HD were studied; representative plots from one HD assay are shown.

**Figure 7:**
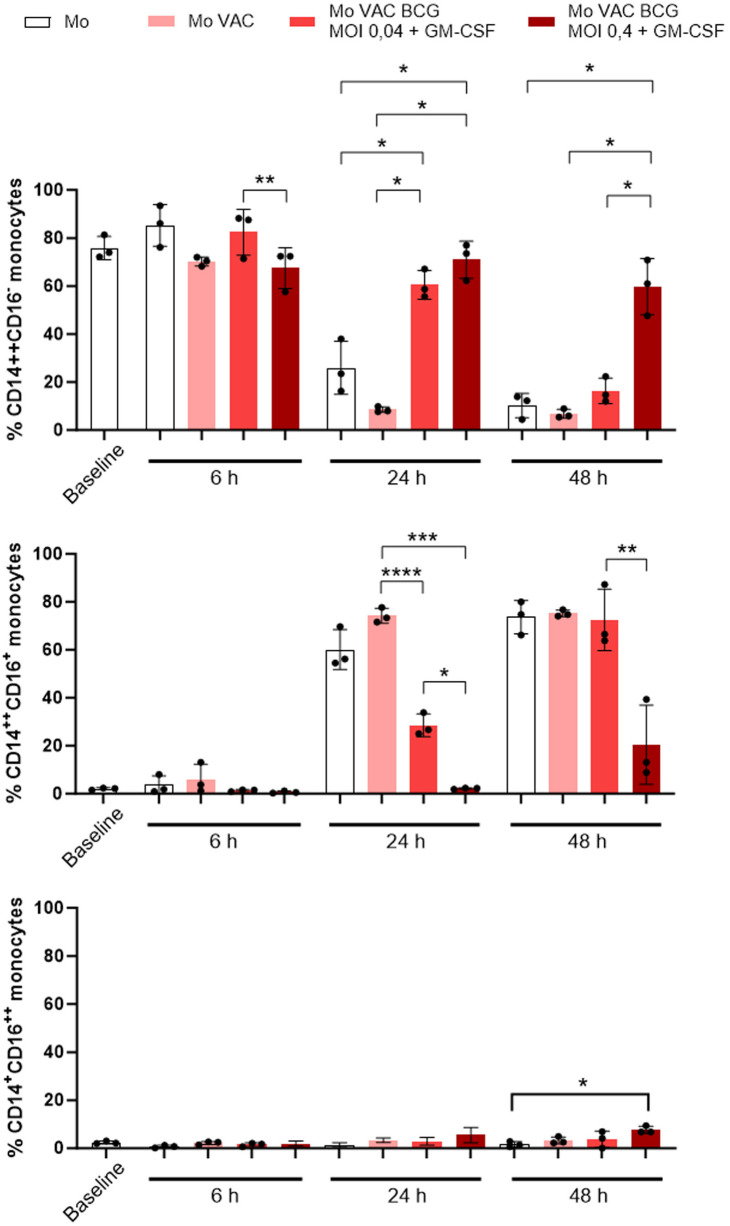
Quantitation of monocytes classes after coculture with VACCIMEL, BCG and GM-CSF. Classical (CD14^++^CD16^-^, upper pannel), intermediate (CD14^++^CD16^+^, middle pannel) and non-classical (CD14^+^CD16^++^, lower pannel) monocytes from HD (n=4), were analyzed by flow cytometry and quantified as described under Materials and methods. Monocytes were cocultured as indicated for 6h, 24h, and 48h, at both BCG: monocyte MOI. Results are shown as mean percentage ± SD. *p<0.05; **p<0.01, ***p<0.001; ****p<0.0001 (GLMs assuming Gaussian errors and Tukey´s multiple comparisons post-test).

We investigated if VACCIMEL uptake by monocytes altered their immunophenotype based on the expression of CD14 and CD16, and if such phenotype was modified by the addition of BCG and GM-CSF. In **Figure 6**, flow cytometry plots of one representative experiment are shown; the analysis of the classical monocytes (CD14^++^CD16^−^), intermediate monocytes (CD14^++^CD16^+^), and non-classical monocytes (CD14^+^CD16^++^) from four independent HD at different times and in the presence of BCG at two different MOI (0.4 and 0.04) and GM.CSF are presented in **Figure 7**. At baseline, and by 6 h of being placed in culture, monocytes displayed the classical phenotype, either with or without VACCIMEL. After 24 h in culture, most monocytes either alone or plus VACCIMEL shifted to the intermediate phenotype (around 70%). This transition was significantly blocked in the presence of BCG (MOI 0.4) and GM-CSF and less inhibited at a BCG MOI of 0.04 and GM-CSF. After 48 h in culture, a slight increase in the intermediate phenotype was observed at MOI 0.4 and an almost complete transition to intermediate phenotype was observed at a BCG MOI 0.04.

Another experiment was carried out with monocytes obtained from a single HD incubated with BCG at two different MOI (0.4 and 0.04) plus GM-CSF for 0, 24 and 48 h. Similar results as those obtained when monocytes incubated with VACCIMEL, BCG and GM-CSF were obtained (**Supplementary Figure 2**).

These experiments suggest that BCG at a 0.4 MOI strongly blocks the transition of classical to intermediate monocyte phenotypes almost completely after 24 h co-culture and that this effect is less pronounced after 48 h. At a lesser BCG MOI (0.04) the same effect, albeit less pronounced, is observed.

### Monocytes cross-present VACCIMEL antigens

**Figure 8:**
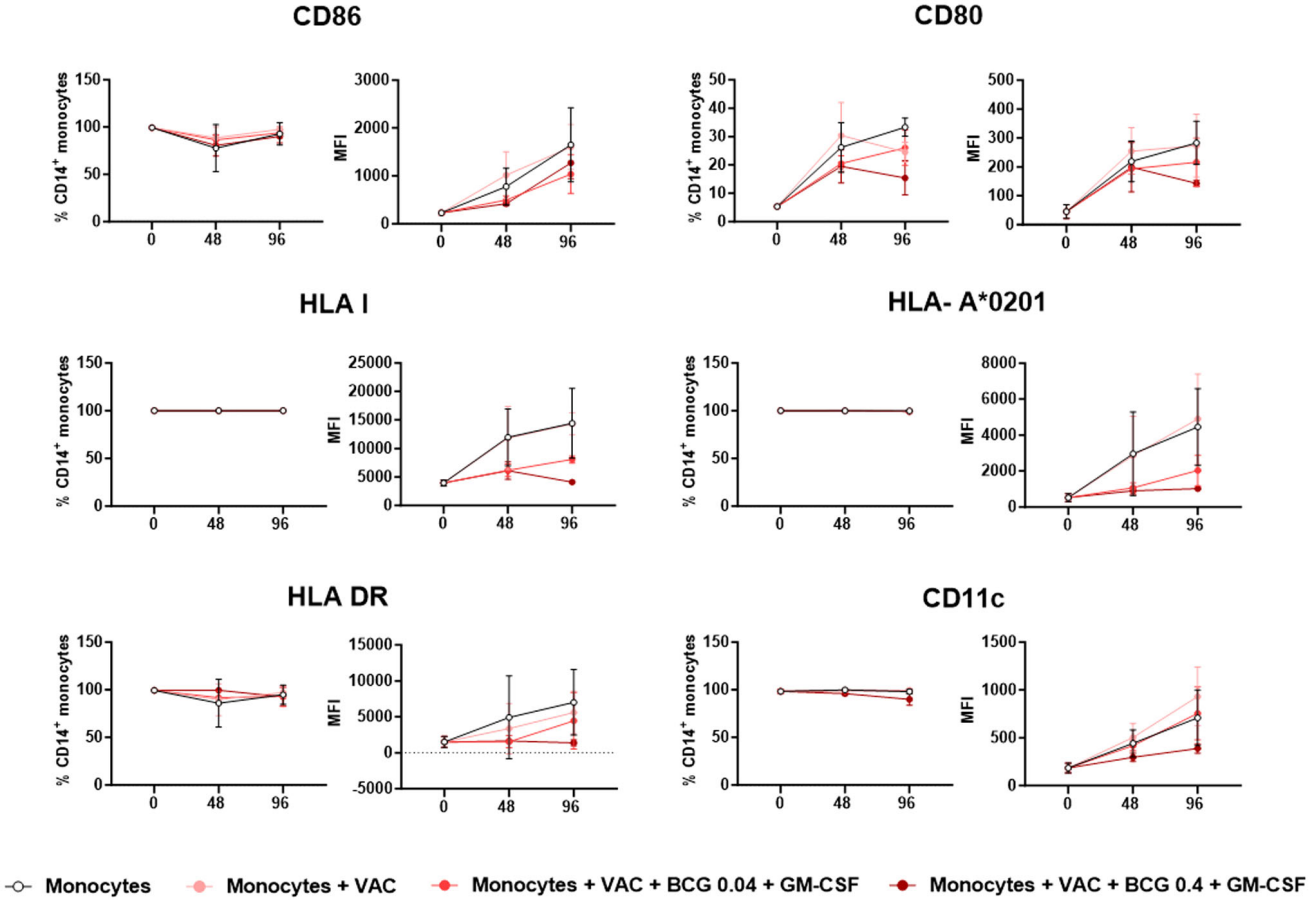
Monocytes expression of markers related to antigen presentation. The expression of specific markers was quantified by flow cytometry after 48 and 96 h co-culture of CD14^+^ monocytes alone, plus VACCIMEL or with BCG (MOI 0.4 or 0.04) and GM-CSF, from 4 HD as described under Materials and Methods. For each marker, the results are shown as the mean ± SD percentage of CD14^+^ monocytes as well as the mean fluorescence intensity (MFI). Statistical analysis is detailed in **Supplementary Table 1** (GLMs assuming Gaussian errors and Tukey´s multiple comparisons post-test).

Antigen presenting cells (APC) may cross-present antigens derived from exogenous antigens to CD8^+^ T cells, loading the cognate peptides in HLA class I molecules. In order to examine whether monocytes share such characteristic, and to which degree, we measured first several markers related to antigen presentation and co-stimulation, such as CD80, CD86, HLA-I, HLA-A*0201, HLA-DR, and CD11c. As observed in **Figure 8**, even in the absence of VACCIMEL or BCG, cultured monocytes at 0 h already expressed the above-quoted markers, although they generally increased after co-culture. Both the percentage of positive monocytes and the mean fluorescence intensity (MFI) were analyzed. Comparisons between the different timepoints in each experimental condition and between the different experimental conditions at each timepoint, are shown in **Supplementary Table 1**, for each marker (GLMs assuming Gaussian errors; Turkey´s multiple comparisons post test). The most remarkable findings were that: i) For CD86, 100% of monocytes expressed the marker, and it significantly increased with culture time; ii) CD80 had a low expression; a significant increase was observed between 0 and 96 h culture, except for monocytes incubated with VACCIMEL plus BCG (MOI 0.4) plus GM-CSF; iii) HLA-I: every cell expressed the marker and at 96 h, incubation with VACCIMEL plus BCG plus GM-CSF significantly diminished MFI at MOI 0.04 (p=0.031) and MOI 0.4 (p=0.025), respectively; iv) HLA-A*0201 similar results that for HLA-I were observed; v) HLA-DR: only the increase in monocytes expression after 96 h of incubation was statistically significant (p = 0.027); vi) CD11c: MFI increased significantly with culture time (0–96 h) in monocytes alone (p=0.0002), monocytes plus VACCIMEL (p<0.0001) and monocytes plus VACCIMEL and BCG at low MOI and GM-CSF (p<0.0001) but did not reach statistical significance at the high MOI.

**Figure 9:**
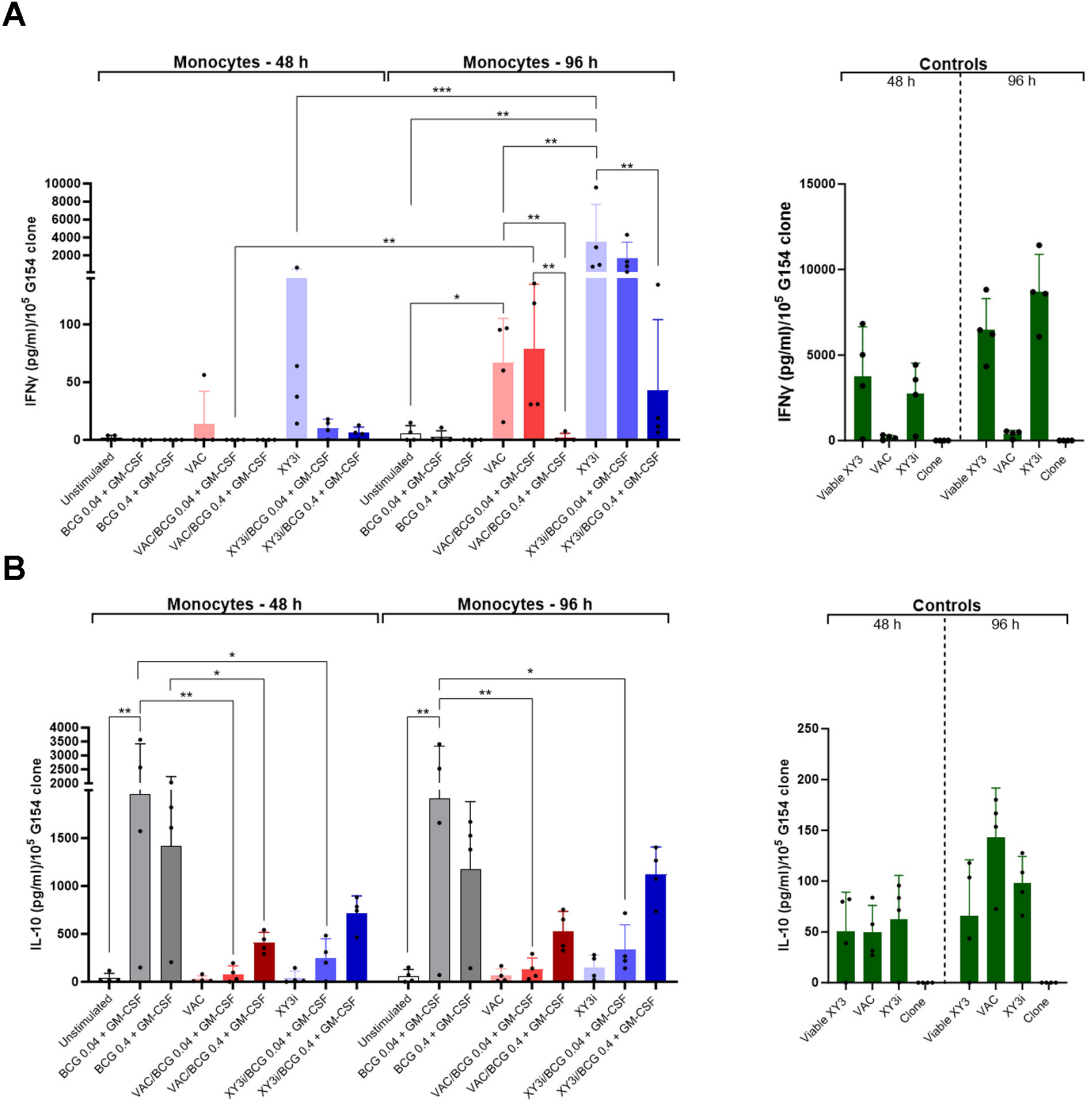
Cross-presentation of gp100/PMEL antigen to G154 clone by monocytes. **(A)** IFN-γ production by G154 clone released to the supernatants after 24 h exposure to monocytes that were previously co-cultured as detailed in the Materials and Methods. The left panel corresponds monocytes purified after 48 h of co-culture with the different antigen sources; also unstimulated monocytes were assayed. The right panel corresponds to the same purified monocytes, which were subsequently cultured for additional 48 h (total incubation time: 96h). Controls of G154 clone incubated with VAC or XY3i or clone left alone (negative control), or viable MEL-XY3 cells (positive control) are also shown (green bars). **(B)** IL-10 production was quantified as described under Materials and Methods, in the same supernatants obtained as above. Results are shown as mean ± SD from four independent experiments. *p<0.05; **p<0.01; ***p<0.001 (GLMs assuming Gaussian errors; Tukey´s multiple comparisons test was used to compare the different experimental conditions at each timepoint; Sidak`s multiple comparison test was used to compare each experimental condition at the different timepoints).

To quantify cross-presentation, a readout of IFN-γ release by the CD8^+^ T cell clone G154, HLA-A*0201-restricted, PMEL/gp100-specific was used. VACCIMEL is a mixture of four allogeneic, irradiated cell lines, two of them without MDA expression. The irradiated MEL-XY3 cell line (MEL-XY3i), one of the cell lines that compose VACCIMEL (VAC) which strongly expresses PMEL/gp100, was used as a positive control. It was investigated if, after 48 h co-culture with VAC or XY3i, monocytes that phagocytosed tumor cells, correctly processed and presented the PMEL/gp100 cognate peptide via HLA-A*0201 to G154 T cells. To avoid possible antigen presentation by remaining tumor cells in the co-culture, monocytes were re-purified after phagocytosis (CD14^+^ purity ranged between 92-97%). Afterwards, purified monocytes were exposed to the G154 clone for 24 h stimulation, and IFN-γ released to the supernatant was quantified by ELISA. In **Figure 9A** (left bars) it may be observed that monocytes loaded with XY3i during 48 h, but not with VAC, were able to cross-present PMEL/gp100 peptide to G154 T cells. Assuming that 48 h were not sufficient to process the phagocytosed antigenic material, purified monocytes were cultured for an additional 48 h after phagocytosis and exposed for 24 h to the G154 clone (**Figures 9A**, right bars). IFN-γ release was significantly increased ~20-fold for MEL-XY3i (p<0.001), and ~5-fold for VAC, probably reflecting that monocytes could achieve at longer times proper antigen processing to degrade PMEL/gp100 to the corresponding peptides, load them onto HLA-A*0201 molecules, and stimulate G154 cells to produce IFN-γ. At both times, VACCIMEL cross-presented PMEL/gp100 directly to G154 cells less efficiently than MEL-XY3i, perhaps due to a smaller relative amount of PMEL/gp100 antigen. Positive control (viable XY3) and negative control (unstimulated G154 clone) were satisfactory.

These results suggest that monocytes can phagocytose PMEL/gp100-containing structures, adequately process them, and cross-present the PMEL/gp100 peptide to T cells.

In the presence of BCG and GM-CSF, PMEL/gp100 cross–presentation to the T cell clone was significantly inhibited (~70-fold; p< 0.01) at the higher BCG MOI, but much less so at the 0.04 MOI, both for VACCIMEL and MEL-XY3i.

Since IL-10 is a potent immunosuppressive cytokine, and based in our results on cross-presentation assays, we evaluated IL-10 production in the monocytes/VACCIMEL co-cultures exposed to G154 T cells (**Figure 9B**). BCG induced a high production of IL-10 by monocytes alone, either at high and low MOI, demonstrating that BCG phagocytosis at both MOI was sufficient to trigger IL-10 production. Interestingly enough, after phagocytosis of VACCIMEL or XY3i cells, IL-10 secretion dampened several folds at the low and high BCG MOI (p<0.05), respectively.

Our results suggest that: i) the VACCIMEL phagocytic process dampens monocyte IL-10 production induced by BCG plus GM-CSF, more so at the lower BCG MOI; ii) IFN-γ production by G154 lymphocytes increases under conditions of lower IL-10 production by monocytes.

## Discussion

The immune response of VACCIMEL-treated patients to most MDA, cancer testis, and neoantigens was usually nil before treatment but increased considerably after several treatment cycles (7, 9). However, the initial steps of the immunization process remain largely unknown. The initial encounter between the main immunogens VACCIMEL and BCG, and the innate immune cells takes place at the dermis. As it refers to BCG, its interaction with the immune system has been extensively studied (19, 20). When BCG is injected into the dermis, several bacillus cell wall components such as peptidoglycans, arabinogalactans, and mycolic acid are recognized by the Toll-like receptors TLR2 and TLR4 present in monocytes (21). Human blood monocytes are found in three different phenotypic states: classical, intermediate, and non-classical, according to their CD14 and CD16 expression pattern. CD14 is a GPI-anchored membrane protein that plays an important role in monocytes, acting as a co-receptor of TLR4 for the recognition of lipopolysaccharide. CD14 also helps to clear apoptotic cells (22), and in fact, we have observed that after VACCIMEL phagocytosis, CD14 translocate from the cell membrane to the monocyte cytoplasm, either in the presence or absence of BCG. In mice, LY6C^low^ monocytes are recruited to sites of infection even earlier than LY6C^hi^ monocytes, through the activation of the CCR2/CCL1 (MCP-1) axis (16), and can participate in the initial inflammatory response by releasing IL-1β, TNF-α, and IL-8 (23). It should be pointed that BCG also triggers the synthesis of IL-6 by monocytes, establishing a systemic response (24).

After 24 h in culture, most classical monocytes shifted to an intermediate phenotype. An unexpected observation, for the first time to our knowledge, was that in the presence of BCG, and using a MOI of 0.4, such transition was completely blocked, although the functional significance of this inhibition has not been explored for the present.

As to the role of GM-CSF as a VACCIMEL component, in a review on GM-CSF, Becker et al. summarized the present evidence on its role under normal or pathological conditions (3). GM-CSF appears to have little influence on the steady-state myelopoiesis, and its levels in circulating blood are practically nil (25). However, its role is important in inflamed tissues, since, in conjunction with IL-4, it is one of the factors that drives the transition from monocytes to monocyte-derived dendritic cells (26). It has also been demonstrated that pathogenic T helper cells produce large amounts of GM-CSF (27), and since most myeloid cells express the GM-CSFR, the interaction between lymphocytes and myeloid cells would lead to an increased inflammatory response. In consequence, we suggest that the addition of GM-CSF to VACCIMEL would play an essential role *in vivo*, fostering the interaction between arriving myeloid cells and lymphocytes at the vaccination site. Also, the well-established role of GM-CSF in the increase of angiogenesis (4, 28, 29) should not be neglected as a mechanism increasing the arrival of myeloid cells to the vaccination site and building a delivery route of melanoma antigens to distant sites.

It should be considered that, besides BCG infection, inflammatory stimuli at the dermis also derive from VACCIMEL itself. With respect to the antigenic load of VACCIMEL, phase contrast microscopy and transmission electron microscopy observations revealed that during the first 48 h in culture, most necrotic and apoptotic cells disintegrated, probably releasing danger-associated molecular patterns (DAMP) signals attracting inflammatory cells (30). Phagocytosis would be triggered by the appearance of “eat me” molecules, such as phosphatidylserine and phosphatidylinositides, on the surface of apoptotic tumor cells, which would be recognized by CD14 present in monocytes with subsequent efferocytosis (31). During the phagocytic process, monocytes emit long pseudopods, which would serve to capture extracellular particles released from dying tumor cells. Globular solid membrane structures also appeared, which established synaptic contacts with apoptotic/necrotic VACCIMEL cells. We suggest that, besides phagocytosis, these structures serve as communication channels between adjacent cells, since small particles of around 100 nm diameter were detected in their lumen. Phagosomes in monocytes appeared, which increased with time from small vesicles to large phagolysosomes, containing cell debris and many of them one to several BCG, and which resolved in approximately 96 h, with the simultaneous increase in the number of lysosomal structures.

A significant objective of this paper was to investigate whether monocytes were able to cross-present captured tumor antigens, and if BCG affected that process. Of note, monocytes expressed relevant molecules associated with antigen presentation machinery, such as CD86, HLA-II, and HLA-I, although CD80 was scarcely expressed. VACCIMEL-derived PMEL/gp100 antigen was poorly cross-presented after monocytes phagocytosis for 48 h, as probed by the exposure to the G154 CD8^+^T cell clone. However, cross-presentation was much higher when monocytes were cultured for 96 h, suggesting that such longer time was required for further enzymatic digestion of phagocytosed material to generate the antigenic peptides. It was found that BCG strongly inhibited antigen cross-presentation, especially at a MOI of 0.4; much less so at 0.04 MOI. As seen in **Figure 8**, the expression of the markers related to antigen presentation also increased at longer culture times. The reasons for the inhibitory role of BCG on cross-presentation remain speculative at the present time.

During this process IL-10, a prototypical pleiotropic immunosuppressive cytokine, could act both on monocytes themselves and on its interaction with lymphocytes. Buozeyen et al. demonstrated that BCG stimulated IL-10 production by macrophages through the suppression of the PI3K/Akt/FOXO3 pathway, FOXO3 being a repressor of IL-10 promoter (32). Riley et al. demonstrated that IL-10 inhibition of TNF-α production required STAT3 and JAK1, attaining maximal efficacy at 100 pg/ml (33). Such inhibitory effect of IL-10 has been also previously demonstrated in monocytes stimulated by poly I: C (34). It has been also previously found that IL-10 blocked the release of TNF-α by monocytes and that such inhibition was abolished by an anti-IL10 antibody (35). Of note, it has been shown that IL-10 does not affect phagocytosis of particulate antigen but decreases antigen processing and T cell priming by downregulating the expression of MHC class II, CD80, CD86, and CD11c in BCG-infected macrophages and DC (36). As to the effects of IL-10 release by monocytes on lymphocytes, Naundorf et al. demonstrated that IL-10 interferes directly with TCR-induced IFN-γ production (37); and Smith et al. showed that IL-10 inhibits T cell function by enhancing N-glycan branching to decrease antigen sensitivity (38).

**Figure 10:**
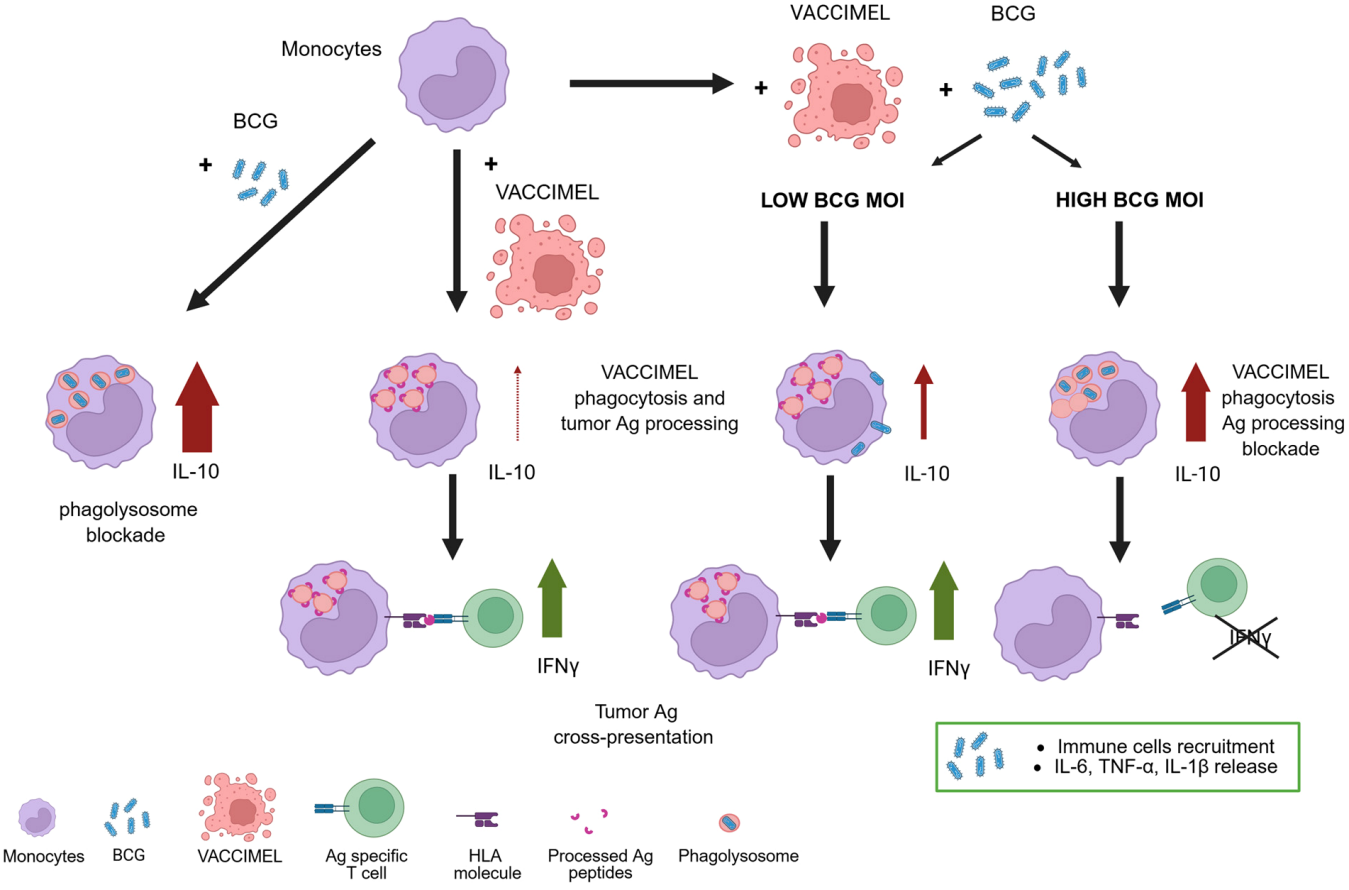
Proposed model of VACCIMEL, monocytes, and BCG interaction. We propose a model in which monocytes, attracted to the vaccination site by BCG, interact with the irradiated melanoma cells comprising VACCIMEL. While monocytes can efficiently phagocytose VACCIMEL, the presence of BCG is expected to trigger IL-10 production. Depending on the local BCG-to-monocyte ratio, antigen processing and cross-presentation of VACCIMEL-derived peptides to T cells may either proceed or be inhibited within monocytes. The precise BCG: monocytes MOI at the inoculation site remains unknown; however, BCG is likely to be cleared primarily by neutrophils that are also recruited to the area. Notably, a balance is established between the strong inflammatory response to BCG—driven by cytokine-mediated immune cell recruitment—and the IL-10-mediated suppression of APC function at the vaccination site. Created with BioRender.com.

In this paper it has been demonstrated that BCG strongly stimulated production of IL-10 by monocytes; strikingly, it has also been found that the simultaneous phagocytosis of BCG and VACCIMEL or MEL-XY3i diminished several folds IL-10 production, to a level enough to allow antigen cross-presentation to proceed, specially at the 0.04 MOI (see **Figure 10** that resumes these findings).

The various aspects linking IL-10 with cytokine production, its relationship with cross- presentation and its inhibition by phagocytosed tumor cells shall be further investigated.

The interaction between VACCIMEL and the immune system is a dynamic process, and we have previously shown in clinical studies that immunity to tumor antigens is built up with successive VACCIMEL treatments (7). Using a different vaccination system, Harris et al. found that the cellular composition of the inoculation site considerably changed after successive vaccinations (39). An important question to pose is whether naïve lymphocytes priming with VACCIMEL antigens occurs at the dermal inoculation site and/or at the draining lymph nodes (LN) or elsewhere. In view of our results, the contribution of monocytes attracted to the inoculation site by the inflammatory signals of the irradiated melanoma cells, BCG, and GM-CSF could be relevant, particularly given that they can efficiently take up VACCIMEL antigens, process and cross-present them to T cells. In fact, we found high numbers of CD11c^+^ cells at the vaccination site, even several weeks after i.d. inoculation (40). These APC probably comprised monocytes, macrophages, and DC. In mice, Rawat et al. demonstrated that monocytes shuttle antigens from the periphery to the draining LN depending on CCL5 produced by DC, and present antigens to naïve cognate T cells (41).

Besides, with each VACCIMEL dose, monocytes would be recruited to the inoculation site along with DC, and, assuming that BCG will be rapidly cleared by multiple cells of the innate immune system, at low MOI. Monocytes that capture, process, and cross-present VACCIMEL-derived antigens after the first shots could contribute to boost already primed circulating T cells at the vaccination site, and expand the anti-tumor immune response with each dose.

The present study has several limitations since it describes the encounter between VACCIMEL and monocytes, only one of the components of the innate immune system. It also remains to be studied if monocytes derived from cutaneous melanoma patients behave similarly to HD.

## Materials and methods

### VACCIMEL and adjuvants

VACCIMEL consists of lethally γ-irradiated cells (apoptotic/necrotic) derived from four cutaneous melanoma cell lines established in-house from human metastatic melanoma tumors. The cell lines MEL-XY1, MEL-XY2, MEL-XY3, and MEL-XX4 were grown in a GMP core facility at the Centro de Investigaciones Oncológicas-FUCA and were cultured as previously described (6). Vaccine preparation was formerly described (7). For all the *in vitro* experiments, lyophilized AJV BCG: Danish 1331 strain was used, which contains 0.75 mg of BCG protein and 2–8 x 10^6^ bacilli. This strain has minimal differences with the Pasteur strain 1173, and both are widely used to vaccinate against tuberculosis (42). Lyophilized recombinant human granulocyte-macrophage colony-stimulating factor (rhGM-CSF, Laboratorio Pablo Cassará, Argentina) was used.

### Number and characteristics of VACCIMEL after thawing

After irradiation and freezing, VACCIMEL cells were thawed and plated in complete melanoma medium (Dulbecco’s Modified Eagle Medium: nutrient Mixture F12 (1:1) (Gibco) supplemented with 2 mM glutamine, 20 nM sodium selenite, 100 μM ascorbic acid, 0.3 mg/ml galactose, 0.15 mg/ml sodium pyruvate and 5 μg/ml insulin, 100 IU/ml penicillin, 10 μg/ml streptomycin plus 10% fetal bovine serum (Natocor, Córdoba, Argentina) for 0, 24, 48, 72, 96 and 168 h and examined under phase contrast microscopy (Olympus CKX41 microscope). Images were digitized (MC190 HD camera, Leica) and analyzed with ImageJ software (NIH). For marker expression determination, 18 x106 irradiated cells were thawed and allowed to recover from the freezing-thawing process in melanoma culture medium for 3 h; this was considered time 0. After that, they were split into three different tissue culture plates, from which at 0, 24, and 48 h, samples were obtained for counting and cell integrity determination by Trypan blue exclusion. The rest of the cells were centrifuged, washed once with PBS, fixed in 10% formalin, and processed for immunohistochemistry.

### Immunohistochemistry

Four micrometers (µm) sections from formalin-fixed, paraffin-embedded VACCIMEL pellets were stained using the following primary mouse anti-human mAbs: Ki-67 (clone 30–9 rabbit monoclonal primary antibody, undiluted, Ventana); PMEL/gp100 (clone HMB45, 1:500, Cell Marque); HLA-I A, B, C (clone EMR8-5, 1:350, Abcam); CD14 (clone EPR3653, 1:300, Abcam); CD11c (clone EP 1347Y, 1:200, Abcam); MLANA/MART-1 (clone A103, undiluted, Ventana) and MAGEA1 (clone SP188, 1:50, Abcam). The avidin–biotin–peroxidase (ABC) system (Vectastain, Vector Labs) was afterward used, and revealed with 3, 3’-diaminobenzidine. Sections were examined by optical microscopy (Olympus BX40 microscope, DP2-BSW software, Olympus), and digitalized pictures were analyzed with ImageJ (43). The percentage expression of each marker was determined by counting 10 fields at 1000× magnification.

### Blood-derived monocytes obtention and purification

Peripheral blood samples (buffy-coats) were obtained from healthy donors (HD) of the Hemotherapy Service of the Instituto Alexander Fleming according to its guidelines and in accordance with the recommendations of the Ethics Committee of the Instituto Alexander Fleming. Peripheral blood mononuclear cells (PBMC) were purified using a Ficoll density gradient (GE Healthcare, UK), and monocytes were purified using an anti-CD14^+^ cell kit (Miltenyi, Germany) following the manufacturer’s specifications. Purified monocytes were cultured in 60 mm Petri dishes without cell adherence treatment, unless specified.

### Uptake of VACCIMEL by monocytes

For these experiments, VACCIMEL melanoma cell lines were stained before γ-irradiation with PKH67 (Sigma- USA) following the manufacturer´s procedure. To evaluate the uptake/phagocytosis of VACCIMEL, 1.2 x 10^7^ purified monocytes were co-cultured with 4 x 10^6^ PKH67-VACCIMEL cells in 5 mL RPMI medium plus 10% FCS, with or without 0.75 mg BCG (MOI 0.4) or 0.075 mg (MOI 0.04) and 100 ng/mL GM-CSF at 37° C, for 0, 6, 24, 48, and 96 h. Also, monocytes alone were equally cultured as controls. At each time point, a control culture performed at 4°C was performed to inhibit the phagocytic process. Afterwards, cells from each culture were stained with FVS510 dye to select a viable cell population and with anti-CD14 and anti-CD16, and analyzed by flow cytometry as described above. To calculate the proportion of blood monocytes that have phagocytosed labeled material from VACCIMEL (**Supplementary Figure 1**), cells were selected as the FVS510 negative population (live cells), then the mononuclear cells were gated and the single cell fraction was selected from the FSC-H vas FSC-A plot, to exclude doublets or steaky cells. These cells were then plotted for CD14-APC and PKH67, and the percentage of PKH67^+^ cells within the CD14^+^ monocytes population was calculated. Remaining cells from the co-cultures were further processed for electron microscopy.

### Transmission electron microscopy

Samples were centrifuged at 1500 rpm, and the pellets were rinsed with wash buffer (0.1 M phosphate buffer, pH 7.4) and then fixed in 1 mL 2.5% glutaraldehyde in 0.1 M phosphate buffer, pH 7.4. The samples were fixed for a minimum of 4 h at 4 °C. Cell pellets were washed twice and post-fixed with 0.5 mL of 1% Osmium Tetroxide (OsO_4_) in 0.1 M phosphate buffer, pH 7.4, at 4 °C for 1 h. After washing and dehydration, samples were embedded in Durcupan. Sections of 0.5 µm were made from the embedded blocks and stained with toluidine blue. A region of the sample was then selected, the block was trimmed with a blade, and ultrathin sections (~ 90 nm) were obtained and mounted onto 300-mesh copper grids. Ultrathin slices were contrasted with Reynolds’ solution, washed, and dried. Grids were analyzed under a Transmission Electron Microscope EM109T TEM (80 kV, Zeiss), and images were acquired with a Gatan ES1000W digital camera. Monocytes that incorporated BCG after 6, 24, and 48 h incubation were quantified from the transmission electron microscopy pictures counting at least 100 cells for each experimental condition, in two independent experiments. Cells were at 3000x magnification. The percentage of monocytes containing BCG (monocytes BCG^+^), either total (Total BCG^+^) or separated into two groups: 1–5 BCG^+^ per monocyte, and >5 BCG^+^ per monocyte were counted.

### Phenotype of monocytes by flow cytometry

To characterize classical, intermediate, and non-classical monocytes, APC anti-CD14 (clone M5E2) and PE Cy7 anti-CD16 (clone 3G8) mAbs were used. Also, CD14^+^ monocytes were stained with PerCp Cy5.5 anti-CD11c (clone B-Ly6), PeCy7 anti-HLA ABC (Clone G46-2.6); BV421 anti-HLA DR (Clone Tu39); APC-H7 anti-CD80 (Clone L307.4); PE anti-CD86 (Clone 2331, FUN-1) and PE anti-HLA-A*0201 (Clone BB7.2). All mAbs were from BD Biosciences. Data acquisition was performed using a FACSCanto II cytometer (BD Biosciences, USA); data analysis was performed with FlowJo software 10.0.7 (Tree Star Inc., USA).

### Antigen cross-presentation assay

G154, a CD8^+^ T cell clone specific for PMEL/gp100 antigen (HLA-A*0201 restricted; KTWGQYWQV) (44), was expanded as previously described (45). CD14^+^ monocytes were purified to >95% from HLA-A*0201 positive HD`s PBMC using anti-CD14 microbeads (Miltenyi Biotec, Germany). Purified monocytes (1.2 x 10^7^ cells) were incubated with VACCIMEL (4 x10^6^ cells), or VACCIMEL (4 x 10^6^ cells) plus BCG (0.4 or 0.04 MOI) plus GM-CSF (100 ng/mL) or alone (unstimulated) in 5 mL RPMI medium plus 10% FBS (culture medium) for 48 h on low adhesion plates to allow antigen uptake. To eliminate VACCIMEL remaining cells and apoptotic bodies after incubation, monocytes were re-purified with anti-CD14 beads as described above and plated (3 x 10^5^ cells) in 0.5 mL culture medium. Also, unstimulated monocytes (3 x 10^5^ cells) incubated only with BCG (0.4 or 0.04 MOI) + GM-CSF were plated. G154 CTL clone (10^5^ cells/0.1 mL) was added and incubated overnight at 37 °C to stimulate IFN-γ release (cross-presentation after 48 h). Also, the same cultures were incubated for additional 48 h (cross-presentation after 96 h) as described above, and then exposed to the G154 clone overnight at 37° C, to stimulate IFN-γ release. In parallel, monocytes were equally incubated with irradiated MEL-XY3 cells (XY3i) expressing PMEL/gp100 antigen in 100% of the cells, instead of VACCIMEL, to assess maximal monocytes cross-presentation under excess PMEL/gp100 antigen. Controls for these experiments included G154 clone incubated with viable MEL-XY3 (10^5^ cells, positive control) or VACCIMEL (10^5^ cells), or XY3i (10^5^ cells), or G154 clone alone (negative control) in the absence of monocytes.

IFN-γ and IL-10 released to the supernatants were determined in triplicate by ELISA (OptEIA IFN-γ; OptEIA IL-10, Pharmingen BD Biosciences, San Diego, CA) according to the manufacturer’s recommendations. Calibration curves were performed for each experiment, and the sample concentration was calculated by log-log regression analysis using GraphPad Prism v9.

### Statistical analysis

Statistical analyses were performed using GraphPad Prism v9 and R software. Total VACCIMEL cell counts and cell integrity at different times after thawing were analyzed using generalized linear mixed models (GLMMs) assuming a gamma error distribution. VACCIMEL antigen expression at different times after thawing, as determined by immunohistochemistry, was analyzed using generalized linear models (GLMs) with a binomial error distribution.

Monocyte phagocytosis of PKH67-labeled VACCIMEL cells was analyzed by GLMMs assuming Gaussian errors, comparing experimental conditions across time points. Phagocytosis of BCG in the presence of GM-CSF was analyzed by comparing the two BCG multiplicities of infection (MOI 0.04 and 0.4) and, within each MOI, across time points. In these analyses, the proportions of monocytes that ingested BCG (BCG^+^), as well as those containing 1–5 bacilli or > 5 bacilli, were modeled using GLMs with a binomial error distribution.

Flow cytometry data for monocyte phenotyping and cross-presentation experiments were analyzed using GLMs assuming Gaussian errors, including the main effects of treatment and time and their interaction. *Post hoc* pairwise comparisons were conducted using estimated marginal means with Tukey or Sidak adjustments for multiple comparisons, as appropriate.

In all analyses, results were considered statistically significant when *p* < 0.05.

## Data Availability

The original contributions presented in the study are included in the article/**Supplementary Material**. Further inquiries can be directed to the corresponding author.
