# Supplementary material for: Unravelling the complexity of the interactions among VACCIMEL, BCG and blood monocytes
Source: Front Immunol. 2026 Feb 26;17:1731270. doi: 10.3389/fimmu.2026.1731270 (PMC12979123; doi:10.3389/fimmu.2026.1731270)
Supplement: Supplementary file 1 [file DataSheet1.docx]

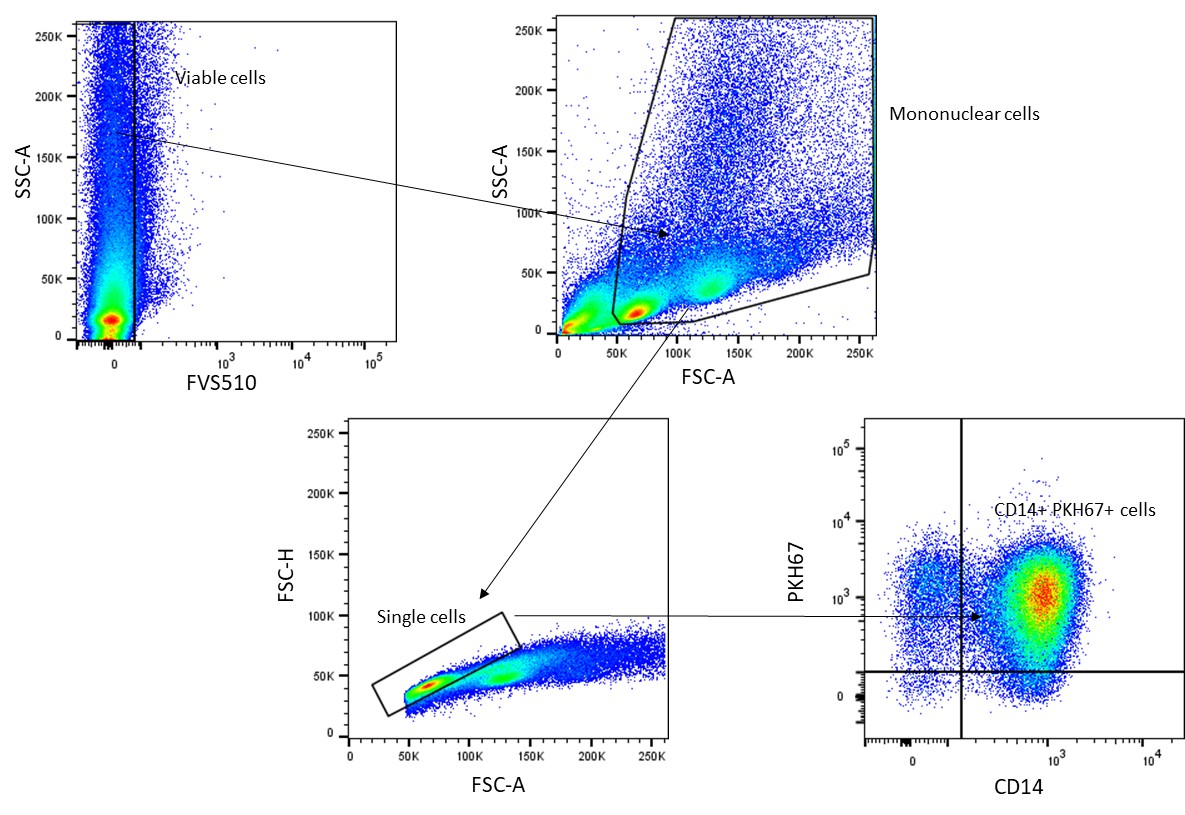


Intermediate monocytes

Non

classical monocytes


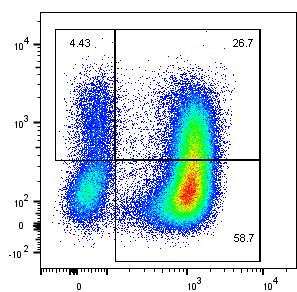


CD16

Classical monocytes

CD14

**Supplementary Figure 1:** Gating strategy to analyze Monocytes uptake of PKH67-labelled VACCIMEL. Live cells were selected as the FVS510 negative population. Then, the mononuclear cells were gated from the SSC-A *vs* FSC-A plot and the single cell fraction was selected from the FSC-H *vs* FSC-A plot, to exclude doublets or sticky cells. These cells were then plotted for CD14 and PKH67, and the percentage of PKH67^+^ cells within the CD14^+^ monocytes population was calculated. Classical monocytes (CD14^++^CD16^−^), intermediate monocytes (CD14^++^CD16^+^), and non-classical monocytes (CD14^+^CD16^++^) were analyzed from the CD14 vs CD16 plot as shown.

.


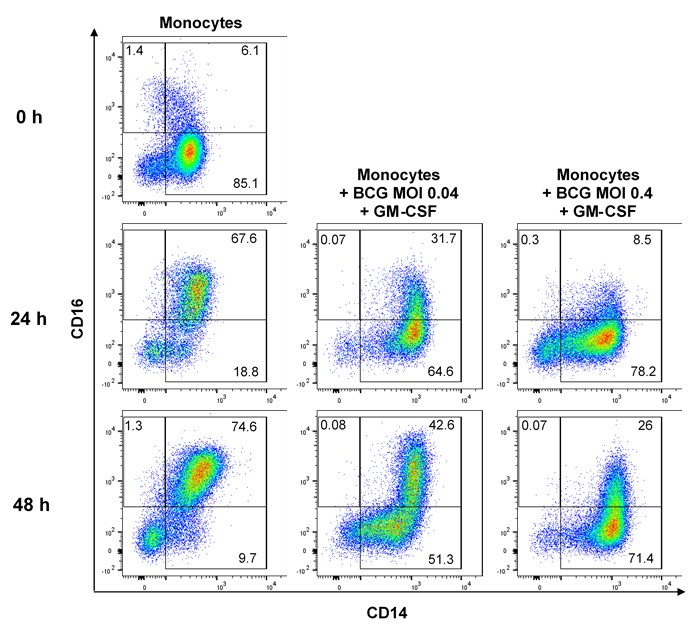


**Supplementary Figure 2. Monocyte phenotype after BCG phagocytosis**. Monocytes from one HD were incubated with or without BCG (MOI 0.04 and 0.4) plus GM-CSF for the indicated times, and their phenotype were analyzed by flow cytometry after labeling of CD14 and CD16 markers as described under Materials and Methods.
